# Mid- to long-wave infrared computational spectroscopy with a graphene metasurface modulator

**DOI:** 10.1038/s41598-020-61998-w

**Published:** 2020-03-25

**Authors:** Vivek Raj Shrestha, Benjamin Craig, Jiajun Meng, James Bullock, Ali Javey, Kenneth B. Crozier

**Affiliations:** 1 School of Physics, University of Melbourne, VIC, 3010 Australia; 2 Department of Electrical and Electronic Engineering, University of Melbourne, VIC, 3010 Australia; 3 Electrical Engineering and Computer Sciences, University of California, Berkeley, Berkeley, CA 94720 USA; 4 Materials Sciences Division, Lawrence Berkeley National Laboratory, Berkeley, CA 94720 USA; 5 Australian Research Council Centre of Excellence for Transformative Meta-Optical Systems, University of Melbourne, Victoria, 3010 Australia

**Keywords:** Optical properties and devices, Metamaterials, Optical spectroscopy, Mid-infrared photonics

## Abstract

In recent years there has been much interest concerning the development of modulators in the mid- to long-wave infrared, based on emerging materials such as graphene. These have been frequently pursued for optical communications, though also for other specialized applications such as infrared scene projectors. Here we investigate a new application for graphene modulators in the mid- to long-wave infrared. We demonstrate, for the first time, computational spectroscopy in the mid- to long-wave infrared using a graphene-based metasurface modulator. Furthermore, our metasurface device operates at low gate voltage. To demonstrate computational spectroscopy, we provide our algorithm with the measured reflection spectra of the modulator at different gate voltages. We also provide it with the measured reflected light power as a function of the gate voltage. The algorithm then estimates the input spectrum. We show that the reconstructed spectrum is in good agreement with that measured directly by a Fourier transform infrared spectrometer, with a normalized mean-absolute-error (NMAE) of 0.021.

## Introduction

Laser beam modulators are ubiquitous components in modern optical systems, with applications that include optical communications, the Q-switching of lasers to generate high energy optical pulses, mode locking and optical beam deflection^1^. Two dimensional arrays of modulator pixels, known as spatial light modulators (SLMs), also find numerous applications, ranging from consumer electronics (e.g. projector displays^2^) to emerging technologies such as holographic displays^2^, optical backplanes^3^, optical information processing^4^, optical tweezers^5^, and single pixel cameras^6^. The above-mentioned applications generally operate from visible wavelengths to the infrared wavelengths used in optical fiber communications (i.e. ~400–1550 nm). Most single-beam modulators and SLMs have therefore been developed for this spectral range. At longer wavelengths, such as the mid- to long-wave infrared (MWIR to LWIR, ∼3–12 *μm*)^7^, modulator technology is generally far less developed, especially for SLMs. This in part due to the fact that materials used in SLMs such as liquid crystals and quantum wells have a response that is highly wavelength-dependent. Materials developed for SLMs in the visible to short-wave infrared are thus generally unsuitable for the MWIR to LWIR. This has motivated investigations into materials and devices suitable for modulators in this spectral range, such as polymer network liquid crystals^8^, quantum wells^9^, quantum cascade laser structures^10^ and graphene^11–14^. The latter has proven to be of much interest. This is partly because it is reasonable to expect that graphene-based modulators could be realized with the areas typical of SLMs (e.g. $\sim 10\,mm\times 10\,mm$), given that fully functional electronic devices (e.g. touch screen panels) have been demonstrated that make use of even larger (30 inch wide) graphene films produced in roll-to-roll processes^15^. In addition to amplitude and phase modulation^11–14,16,17^, applications such as polarimetry^18^, motion sensing^19^, and single pixel imaging^20^ have been demonstrated using graphene-based metasurface devices at MWIR-LWIR wavelengths. While promising results were obtained in these previous works, one of the most important applications of the MWIR-LWIR spectral range is that of spectroscopy. This plays a vital role in areas that include biology^21^, medicine^22^, chemical analysis^23^, forensic science^24^, and food safety testing^25,26^. The conventional workhorse for MWIR-LWIR spectroscopy is the Fourier transform infrared spectrometer (FTIR). The light to be analyzed enters the system, passes through an interferometer (e.g. Michelson) and then is recorded by a photodetector. One may think of the interferometer as a modulator, whose tunable transmission spectrum allows the system to determine the spectrum of the input light. This motivates an investigation into performing MWIR-LWIR spectroscopy using another type of modulator, i.e. a graphene-based metasurface, that is considerably smaller than the interferometers used in FTIR systems. This is the topic of this work.

Here, we experimentally demonstrate graphene metasurface modulators, each composed of metallic nanostructures and a dielectric cavity integrated with graphene, whose MWIR-LWIR reflection spectra can be tuned via a voltage (*V*_G_) applied to its back gate. Each modulator has an active region with an extent of 30 μm × 30 μm. We demonstrate an array of three such devices, each of which is tailored to operate in a different portion of the MWIR-LWIR band. In principle, combining these modulators with matching detectors would result in a very compact system for computational spectroscopy. We demonstrate this principle using our metasurface devices. We illuminate our devices with the unknown spectrum, and record the signal measured by a detector (representing total reflected power) at different *V*_G_ values. This information is then provided, along with the device characteristics (i.e. reflection spectra at different *V*_G_), to an algorithm that estimates the (previously unknown) input spectrum. For experimental convenience, these measurements are performed using an FTIR microscope (rather than three matching detectors). It should be noted however the spectra recorded by the FTIR are integrated (over wavelength) to give total reflected power (vs gate voltage), to mimic what would be recorded by matching detectors of an integrated system. We demonstrate the reconstruction of the spectrum of an infrared light source comprising the silicon carbide globar of our FTIR system. We show that the spectrum reconstructed in this way is in good agreement with that measured directly by our FTIR system.

## Design and Simulation

**Figure 1:**
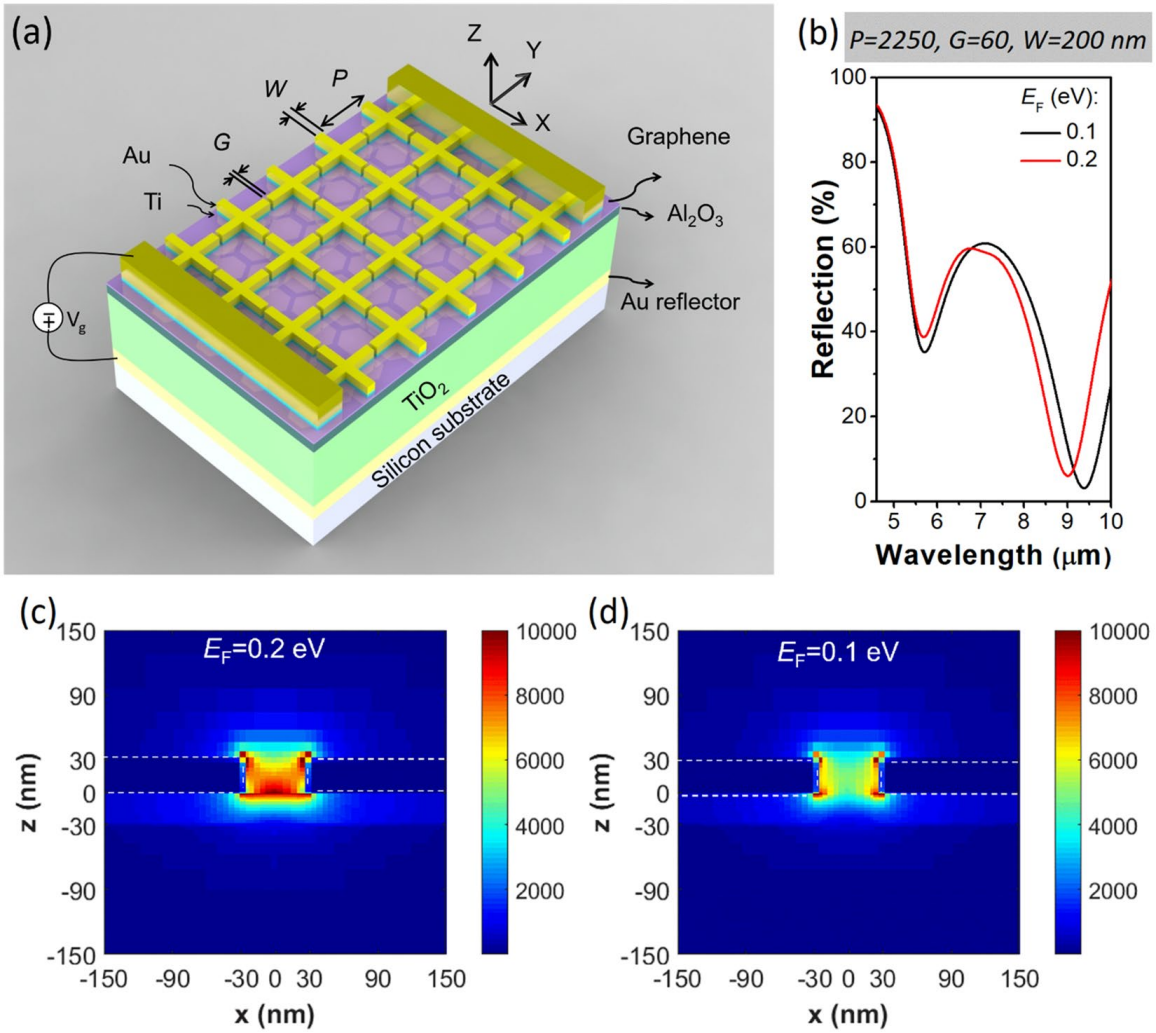
(**a**) Schematic of graphene metasurface modulator. (**b**) Simulated reflection spectra of device for graphene Fermi energies of 0.1 eV and 0.2 eV. Simulated electric field intensity(|E/E_0_|^2^) distribution in cross-section of device in xz-plane, bisecting antennas. Illumination is from air side at normal incidence with E-field polarized along x direction, at a wavelength of λ = 9009 nm. Fermi energy of graphene is (**c**) 0.2 eV and (**d**) 0.1 eV.

A schematic diagram of our metasurface modulator is shown as Fig. 1a. It comprises a periodic array of cross-shaped nanoantennas formed over a single layer of graphene which in turn sits on a stack containing alumina (Al_2_O_3_), titanium dioxide (TiO_2_), and gold (Au) layers on a silicon substrate. The operating principle is as follows. The Au layer acts as a back gate, with the Al_2_O_3_/TiO_2_ layers serving as the gate dielectric. Applying a voltage *V*_*G*_ to the back gate changes the areal free carrier density in the graphene approximately according to $n\approx {C}_{g}\Delta V/e$ where $\Delta V={V}_{G}-{V}_{CNP}$ and *C*_*g*_ is the gate capacitance per unit area^19^. Here, ${V}_{CNP}$ denotes the voltage of the charge neutrality point, at which the graphene has maximum resistance. The carrier density is related to the Fermi energy as follows^19^: ${E}_{F}=\hslash {v}_{F}\sqrt{\pi n}$, where ${v}_{F}=1\times {10}^{8}\,cm/s$ is the Fermi velocity. The optical response of graphene can be modeled by considering it as a surface current whose magnitude is given by the product of the electric field and the optical conductivity^27^. The latter is a function of the Fermi energy, e.g. as given by the random phase approximation in the local limit^27^. Application of the gate voltage thus modifies the optical properties of the graphene, enabling the metasurface to be electrically-tunable. As graphene is atomically thin, however, its interaction with light is comparatively weak. We thus integrate it with the plasmonic antennas, the dielectric cavity, and a metal (Au) reflector. This configuration in principle allows critical coupling, in which all of the light incident upon the structure is absorbed^11,17^. The structural parameters of the device include the nanoantenna period (*P*), width (*W*), gap (*G*), and the thicknesses of the dielectric layers $({t}_{Ti{O}_{2}},\,{t}_{A{l}_{2}{O}_{3}})$. These are schematically illustrated as Fig. 1a. Simulated reflection spectra for a device with parameters $P=2250\,nm$, $W=200\,nm$, $G=60\,nm$, ${t}_{Ti{O}_{2}}=800\,nm$ and ${t}_{A{l}_{2}{O}_{3}}=30\,nm$ are shown in Fig. 1b. To mimic electrostatic doping via a gate voltage, simulations are performed for two different values of the Fermi energy. It can be seen that varying *E*_*F*_ from 0.1 to 0.2 *eV* results in the spectral position of the reflection dip blue-shifting from 9383 nm to 9009 nm. In Fig. 1c,d, we plot the simulated electric field intensity in a cross section (*xz* plane) that bisects the antenna structure. These simulations are performed for light normally-incident upon the device at a wavelength of $\lambda =9009\,nm$. It can be seen that the field enhancement in the gap is greater for ${E}_{F}=0.2\,eV$ (i.e. Figs 1c and S1a) than for ${E}_{F}=0.1\,eV$ (i.e. Figs. 1d and S1b). This is consistent with the far-field simulations (Fig. 1b).

One of the challenges associated with the dielectric spacer/metal reflector approach at mid- to long-wave infrared wavelengths is that the optical designs will often call for the dielectric spacer to be relatively thick. The dielectric spacers used in the fabricated devices in refs. ^11,17,20,28^, for example, were hundreds of nanometers thick. This lowers the capacitance of the device, meaning that high bias voltages are necessary to control *E*_*F*_. Zeng *et al*.^29^ addressed this by forming the dielectric spacer from thin (6 nm) and thick (400 nm) films of Al_2_O_3_ and amorphous silicon (a-Si), respectively. The a-Si was optically transparent at the wavelength of operation, yet electrically conductive, allowing the device to have the high capacitance associated with the thin Al_2_O_3_. Lee *et al*.^30^ addressed this issue using a different approach via forming the gate dielectric from a material with a high permittivity (tantalum pentoxide, ${\varepsilon }_{r}=22$). The efficacy of this technique motivates us to investigate whether there are other materials with high dielectric constants and infrared transparency that might also be suitable. It has been reported^31^ that, depending on crystal structure and method of deposition, titanium dioxide (TiO_2_) can have a relative permittivity of 80–110. Here we demonstrate that graphene metasurface modulators based on TiO_2_, with the addition of Al_2_O_3_ to mitigate gate leakage, enable the use of relatively modest gate voltages. We anticipate that this will be advantageous not only for the spectral reconstruction application we report, but also for other applications of mid- to long-wave infrared modulators based on graphene.

## Fabrication and Characterization

**Figure 2:**
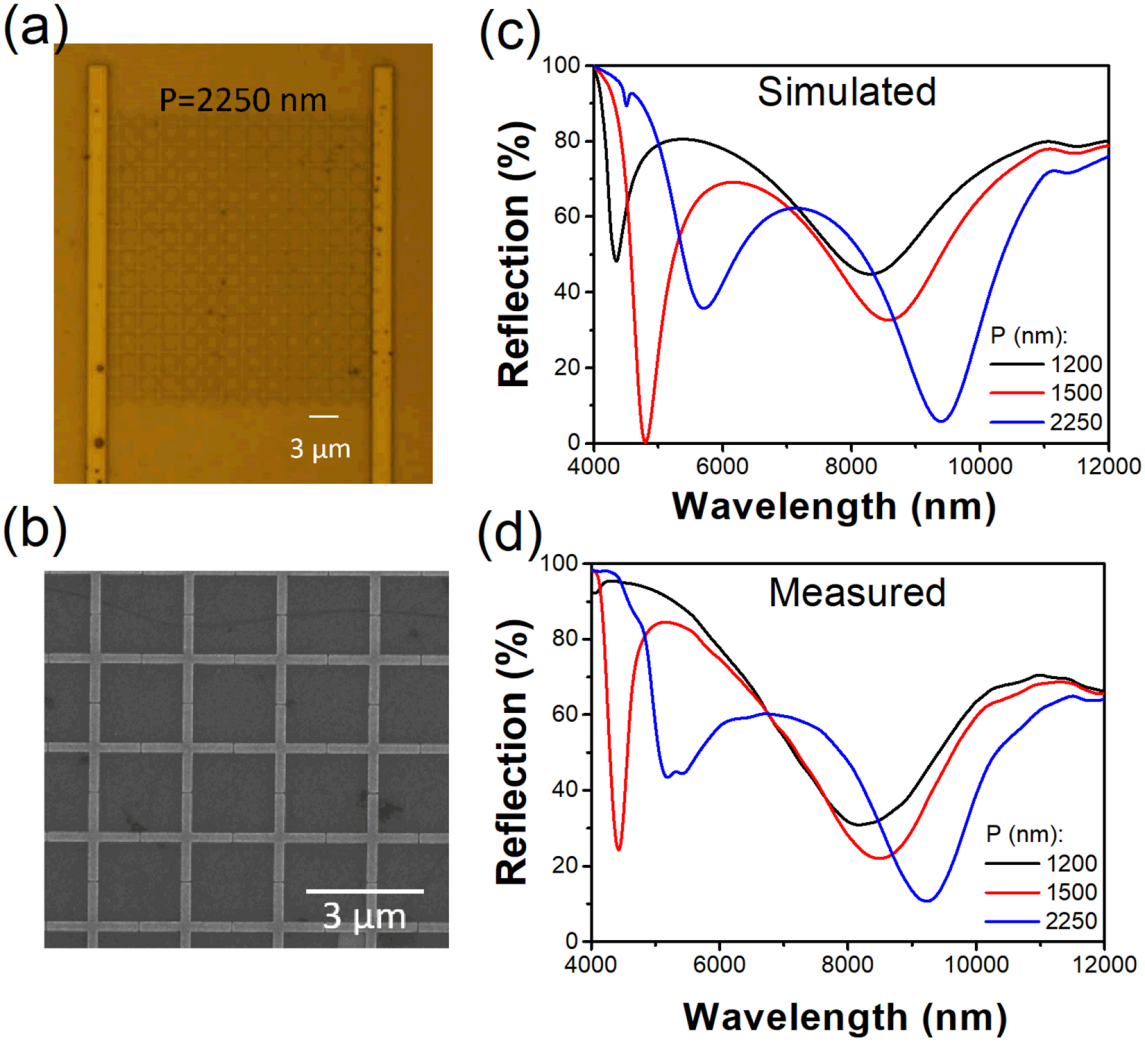
(**a**) Optical and (**b**) scanning electron microscope images of device for which plasmonic antennas have period P = 2250 nm. (**c**) Simulated and (**d**) measured reflection spectra of devices with periods of P = 1200, 1500 and 2250 nm.

We fabricate our graphene metasurface modulators via a series of thin film deposition steps, graphene transfer and electron beam lithography. The latter is used to define the graphene channels, antenna structures and the metal electrodes. The thin film depositions include electron beam evaporation (for Au and Ti), reactive sputtering (for TiO_2_) and atomic layer deposition (for Al_2_O_3_). A wet transfer technique is used to transfer the graphene from a copper foil to the device. The fabrication process is described in greater detail in the Methods section. Devices containing arrays of antennas with periods *P* of 1200, 1500 and 2250 nm are fabricated. An optical microscope image and a scanning electron micrograph (SEM) of the device with plasmonic antennas with period $P=\,2250\,nm$ on the top of the graphene sheet are shown as Fig. 2a,b, respectively. Optical microscope and SEM images of the other two devices (plasmonic antennas with periods $P=\,1200\,nm$ and $1500\,nm$) are included in the Supporting Information (Fig. S2). From the SEM images of Figs. 2 and S2, it can be seen that the antenna structures are accurately defined. We next measure the reflection spectra of the fabricated devices using an FTIR microscope (Spotlight 200i, Perkin Elmer). These are normalized to the reflectance measured from a reference sample comprising a gold film. The reflection spectra are also simulated using the FDTD method. The results are shown as Fig. 2c,d. It can be seen that the simulated and measured reflection spectra are in good agreement. We also note that our device is inherently polarization-insensitive for normal incident illumination. This differs from previous approaches^11–14,16–20,28,29^ in which asymmetric structures were employed (that are inherently polarization-sensitive).

**Figure 3:**
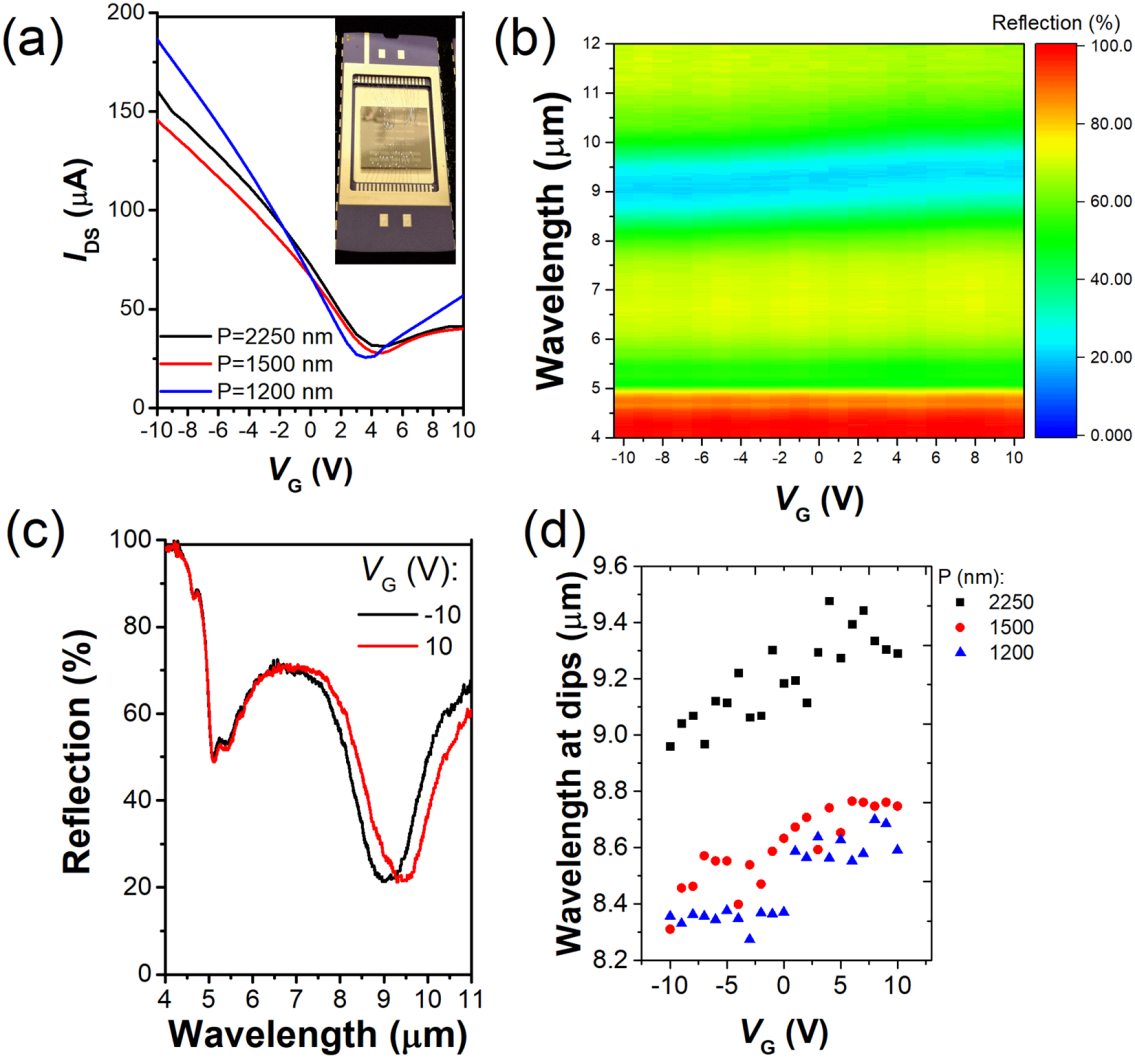
(**a**) Measured drain-source current *I*_*ds*_ vs gate voltage *V*_*G*_ at drain-source bias ${V}_{DS}=100\,mV$ for devices with different antenna periods *P*. Photograph of device after being wire-bonded to chip carrier is shown in the inset. (**b**) Colormap plot of measured reflection spectrum of device with P = 2250 nm vs gate voltage (${V}_{G}\,=-\,10\,V$ and $10\,V$ in steps of 1 *V*). (**c**) Measured reflection spectra of sample with period $P=\,2250\,nm$ at ${V}_{G}\,=-\,10\,V$ and $10\,V$. Color represents reflectance. (**d**) Plot of wavelength at which measured reflectance is minimum for each gate voltage for devices with different periods P = 2250 nm (black squares), 1500 nm (red dots) and 1200 nm (blue triangles).

We next discuss the electrical characterization of our devices. The sample chip is mounted in a chip carrier. The contact pads of the devices are then wire bonded to the pins of the chip carrier (Fig. 3a). Our devices comprise field effect transistors and we thus refer to the two contacts to the graphene (Fig. 1a) as source and drain electrodes. We next characterize the three devices electrically by measuring the drain-source currents IDS as a function of gate voltage *V*_*G*_. In these measurements, the drain-source bias *V*_*DS*_ is set to 100 mV. The results are shown as Fig. 3b. These display the features typical of graphene FETs. It can be clearly seen that gate voltage required to reach the graphene charge neutrality point (*V*_*CNP*_), at which IDS reaches a minimum, is a few volts (<5 V) for all three devices. Next, we measure reflection spectra ${R}_{VG}(\lambda )$ of the three devices at 21 different values of *V*_*G*_, ranging from −10*V* to 10*V* in steps of 1*V*. This is performed with our FTIR microscope using unpolarized light, and the results are again normalized to the reflection measured from a gold film. Results are shown for the device with $P=2250\,nm$ as Fig. 3b,c, while those for the devices with $P=1200\,nm$ and $1500\,nm$ are shown in the Supporting Information (Fig. S3). From Fig. 3c, it can be seen that varying *V*_*G*_ from −10*V* to 10*V* for the $P=2250\,nm$ device results in a substantial shift in the position of the reflection dip. Fig. 3b plots a colormap of gate-dependent reflection spectra at each of 21 different gate voltages. This is further quantified by Fig. 3d which plots the wavelength at which measured reflectance is minimum for each of 21 gate voltages for three devices with different periods different periods P = 2250 nm (black squares), 1500 nm (red dots) and 1200 nm (blue triangles). It is observed that varying *V*_*G*_ from −10*V* to 4*V* (i.e. near V_CNP_), results in the reflection dip shifting from $\lambda \,=\,8.961$ to 9.477 μm, i.e. representing a tuning span of $516\,nm$. As the gate voltage is further increased $({V}_{G}\,=\,10\,V)$, the reflection dip shifts to $\lambda \,=9.291\,{\rm{\mu }}{\rm{m}}$. Similarly, for the $P\,=\,1500\,nm$ device, the reflection dip shifts from $\lambda =8.31108\,{\rm{to}}\,8.74119\,{\rm{\mu }}{\rm{m}}$ as *V*_*G*_ is varied from −10*V* to 6*V* (again near V_CNP_), i.e. a tuning span of $454.11\,nm$. For the device with $P=1200\,nm$, the reflection dip shifts from $\lambda \,=8.357$ to $8.637\,\mu m$ as *V*_G_ is varied from −10*V* to 3*V* (near Dirac voltage), i.e. a tuning span of 280 *nm*. It is worth noting that the wavelength shift per applied voltage with our devices is as large as 36.56 which surpasses the results of refs. ^11,14,16,17,29^, as seen in Table 1 below. We also note although the wavelength shift per voltage in the device reported by Zeng *et al*.^29^ is 2.7 times better than our work, our work is polarization-insensitive which is not the case for refs. ^11,14,16,17,29^ which are inherently polarization-sensitive. To further characterize our devices, we calculate the wavelength dependent modulation depth (i.e., $1-\,{\rm{\min }}[{R}_{VG}(\lambda )]/\,{\rm{\max }}\,[{R}_{VG}(\lambda )]$, where ${\rm{\max }}\,[{R}_{VG}(\lambda )]$ and ${\rm{\min }}\,[{R}_{VG}(\lambda )]$ are the maximum and minimum achievable reflection values, respectively, at a given wavelength *λ*. This information is extracted from the reflection spectra that are measured at different gate voltages. The results are shown as Fig. S4. The results show that the maximum modulation depth for the device with P = 2250 nm is 35.5% at *λ* = 9.67 μm, while it is 20.63% at *λ* = 8.99 μm (for device with P = 1500 nm) and 18.6% at *λ* = 8.95 μm (for device with P = 1200 nm). Considering all the three devices together, the range of tuning is from $\lambda \,=8.311\,{\rm{to}}\,9.477\,{\rm{\mu }}{\rm{m}}$, thus providing a total tuning span of $1166\,nm$. This tuning span is not gap-free, but this does not prevent the demonstration of spectral reconstruction with our devices. We anticipate that this large tuning range coupled with appreciable modulation depth over the wavelength range of interest enabled by our devices would be helpful for spectral reconstruction since it allows us to create different spectra^32^ by controlling the gate voltage.

**Table 1:** Wavelength shift per unit voltage of graphene-based mid- to long-wave infrared modulator devices.

Reference	Voltage range	Wavelength shift (nm)	Wavelength shift per voltage (nm/V)	Polarization- insensitive?
11	0 to 80 V	600	7.5	No
14	0 to −390V	330	0.846	No
16	90 to −80V	490	3.267	No
17	0 to 183 V	<1000	<5.464	No
29	+7 V to −3V	1 μm	100	No
Our work	−10 to +4 V	516	36.85	Yes

## Spectral Reconstruction Demonstration

We next demonstrate spectral reconstruction in the mid- to long-wave IR using our metasurface device. As discussed, this spectral range is of much interest due to numerous medical, industrial and scientific applications^21–26^. The workhorse tool for spectroscopy in a laboratory setting in this spectral range is the FTIR spectrometer. For some field-based applications of portable spectrometers, the size and weight of the spectrometer are much more important than its resolution. These applications include rapid safety screening and threat detection^33^ (e.g., homeland security, airport screening, first response, environmental, home and workplace safety); law enforcement (e.g., identification of narcotics^34^ and other illegal materials); and rapid purity or authenticity determination (product safety and adulteration detection, product authenticity and anti-counterfeiting^35,36^, point-of-use testing in processes, and raw material identification^37^). This has motivated investigations into alternative approaches to mid- to long-wave IR spectroscopy. At visible wavelengths, it has been shown that an array of spectral filters integrated with an array of photodetectors, used in conjunction with an appropriate algorithm, enables the realization of a very compact spectrometer^32,38^. In the mid- to long-wave IR, this approach has been investigated in refs. ^39,40^ using plasmonic nanostructures as the filter array. In those works, however, a relatively large number of filters were employed, i.e. 30 in ref. ^39^ and 116 in ref. ^40^. This was in part due to the fact that the filters employed were not tunable, i.e. they had fixed filtering characteristics. Here, we demonstrate spectral reconstruction using our graphene metasurface modulators. That only three filtering elements (i.e. graphene metasurface modulator devices) are required is due to the fact that each has a voltage-tunable spectral response. We note that a voltage tunable photoresponse has been shown using other structures, e.g. with Ge nanowires^41^. It may be interesting to also demonstrate computational spectroscopy using such structures.

**Figure 4:**
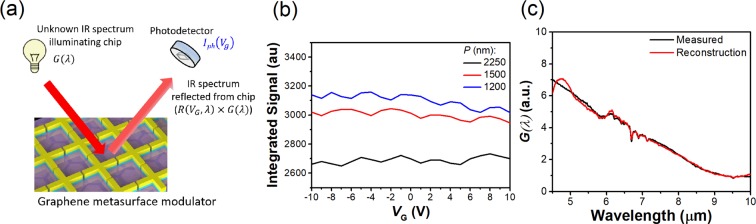
(**a**) Schematic illustration of principle of spectral reconstruction with graphene metasurface modulator. (**b**) Integrated signal vs gate voltage for three metasurface devices. (**c**) Spectra of infrared light source ($G(\lambda )$, from silicon carbide globar) as reconstructed using graphene metasurface modulator (red curve) and as measured directly by FTIR (black curve). Wavelength range is $\,\lambda =4.5\,\mu m$ to $10\,\mu m$, with 2751 data points.

The intended mode of operation is illustrated schematically as Fig. 4a. The unknown spectrum *G*(*λ*) illuminates the device, with a photodetector used to measure the reflected power as a function of gate voltage *V*_*G*_. This information (i.e. photocurrent vs gate voltage, ${I}_{ph}({V}_{G})$) is then provided to an algorithm, along with the measured reflection spectra of the device at different gate voltages ${R}_{VG}(\lambda )$. The algorithm then estimates the incident spectrum. As a proof-of-principle demonstration, we reconstruct the spectrum of the light source of our FTIR system (silicon carbide globar). This is performed as follows. For experimental convenience, we use the microscope of the FTIR system to measure the light reflected from our metasurface devices as a function of gate voltage *V*_*G*_. The FTIR system provides this information in the form of spectra, each representing $(\lambda )=G(\lambda )\times MC{T}_{resp}(\lambda )\times {R}_{VG}(\lambda )$, where G(*λ*) is power density spectrum of the globar source and $MC{T}_{resp}(\lambda )$ is responsivity of the mercury cadmium telluride (MCT) detector of the FTIR microscope. $MC{T}_{resp}(\lambda )$ was determined by dividing the incident spectra measured by the MCT normalized to that measured by the internal deuterated triglycine sulfate (DTGS) in the FTIR, which has a wavelength-independent responsivity. The reflected spectra are normalized to the reflection from a gold reference to obtain ${R}_{VG}(\lambda )$. To emulate the signal that would be collected by the configuration schematically illustrated as Fig. 4a, we integrate each spectrum (*λ*) over the wavelength range $\lambda =4.5\,\mu m$ to $\,10\,\mu m$. As there are three devices, each measured with 21 different values of gate voltage *V*_*G*_, this yields a column vector (63 × 1) that mimics what would be measured by three detectors matched to the three metasurfaces. This data is shown as Fig. 4b. This column vector is input, along with RVG(*λ*), to a recursive least squares (RLS) algorithm^42^ to estimate $G(\lambda )$, i.e. the spectrum of our globar. The results are shown in Fig. 4c. It can be seen that the reconstructed spectrum is in good agreement with the spectrum measured directly by our FTIR system. We note that while the spectral dip of our metasurface devices can be tuned from $\lambda \,=\,8.31\,\mu m$ to 9.47 *μm* the reconstruction is performed over a much wider range (λ = 4.5 *μm* to 10 *μm*). This is because the reflection spectra of the devices are uniquely modified over an extended wavelength range.

We next investigate how the reconstruction varies with the number of metasurface devices used. This is done by performing reconstructions with one, two and three devices. The results are shown in the Supporting Information (Fig. S5). Figure S5a shows the signals measured from the three different devices vs gate voltage. Note that this plot is the same as Fig. 4b and is included in the Supporting Information for convenience. In Fig. S5b–d, reconstructions (of $G(\lambda )$) are presented that are obtained using one device $(P=2250\,nm)$, two devices ($P=1500\,nm$ and $2250\,nm$), and three devices ($P=1200\,nm$, $1500\,nm$ and $2250\,nm$), respectively. As before, these are performed over the wavelength range $\lambda =4612.2\,nm$ to $10072.8\,nm$, with 1917 data points per reconstruction. The accuracy of reconstruction is quantified by calculating the normalized mean-absolute-error (NMAE), which is the mean of the absolute difference between the spectrum measured by the FTIR and that reconstructed by our system, normalised to the peak signal value. It can be seen that the reconstruction improves as the number of devices employed is increased, with the NMAE value decreasing from 0.093 when only device (with P = 2250) is used for reconstruction, to 0.031 when two devices (with P = 2250 and 1500 nm) are used. The NMAE further decreases to 0.021 when all three devices (with P = 2250, 1500 and 1200 nm) are used for reconstruction. It is to be noted that despite of some fluctuations in the position of the dips (Fig. 3d), the reconstructed spectra are in good agreement with the measured spectra. We attribute such fluctuations in the position of the dips to the hysteresis (Fig. S6) of the device resulting from the charge transfer from neighboring adsorbates (such as water molecules) or charge injection into the trap sites on the dielectric substrate.

We next check the robustness of this method by re-doing the reconstruction with a delay of several months between the characterization of the metasurface stored in vacuum desiccator (i.e. determination of ${R}_{VG}(\lambda )$) and the measurement of the signal vs gate voltage (i.e. collection of data when metasurface is illuminated by unknown spectrum). These results are obtained with the $P=2250\,nm$ device over the spectral range $\lambda =7501.75\,nm$ to $10003\,nm$. The results are shown in Fig. S7. In Fig. S7a, we also show the reconstruction obtained as before (i.e. without the delay). In Fig. S7b, we show the results obtained when the signal from the metasurface device vs gate voltage is measured several months (150 days) after the measurements of the reflection spectra ${R}_{VG}(\lambda )$. It can be seen that both reconstructions (Fig. S7a,b) are in reasonable agreement with the spectrum measured directly by our FTIR system.

## Discussion and Conclusions

In summary, we demonstrate the concept of spectral reconstruction using graphene metasurface modulators. Three electrically tunable graphene metasurfaces, each comprising metallic nanostructures of different periods and a subwavelength cavity integrated with graphene, are fabricated and characterized. When considered together, these devices enable the spectral position of the reflection dip to be tuned over the range $\lambda =8.31\,\mu m\,{\rm{to}}\,9.477\,\mu m$ We demonstrate the spectral reconstruction over the wavelength range from $\lambda =4.5$ to $10\,\mu m$ using our devices in combination with a recursive least squares (RLS) algorithm. The demonstrated metasurface based IR modulator could be combined with a detector to realize a miniaturized system for IR spectroscopy. We anticipate that this approach might find applications for which very compact and lightweight IR spectrometers are needed, such as in hazardous gas detection and environmental monitoring. In this work, our metasurface modulator is operated in reflection mode. While it would be possible to combine our (reflection-mode) device with a detector to realize a compact spectrometer, an even smaller system would result via a transmission-mode device.

## Methods

### Electromagnetic simulations

Three-dimensional simulations are performed using the finite difference time domain (FDTD) method with a commercial software package (Lumerical). Graphene is modelled as an infinitesimally thin conductive surface, whose surface conductivity is as given in ref. ^27^. The frequency-dependent complex refractive indices of Au, Ti, TiO_2_, Al_2_O_3_, SiO_2_ and Si used in the simulations are taken from the compilation by Palik^43^.

### Sample fabrication

Fabrication of our device starts with the deposition of Au (120 nm thick), TiO_2_ (800 nm thick) and Al_2_O_3_ (30 nm thick) layers on a highly doped silicon wafer. We next transfer a CVD graphene monolayer (grown by chemical vapor deposition on Cu) onto the sample using a poly methyl methacrylate (PMMA) transfer technique. For this, a layer of PMMA is spin-coated onto a piece of graphene on Cu to form a support for graphene and placed in ammonium persulfate (NH_4_)_2_S_2_O_8_ solution for 3 hours to etch away the Cu foil, after which the PMMA/graphene stack is placed into deionized water and rinsed. The PMMA/graphene stacked is then scooped from the water to our desired substrate. The PMMA (on the graphene) is removed by placing the sample in acetone (60 °C) and sample is then washed with iso-propanol. It is then blown-dry with a nitrogen gun, thus completing the graphene transfer. PMMA (from Microchem, with molecular weight 495 K) is then spun on to the sample to a thickness of 200 nm. The PMMA is then exposed by e-beam lithography (100 keV, Vistec EBPG5000plusES) to define the nanoantenna arrays. The exposed PMMA is developed in a mixture (3:1) of isopropanol:methyl isobutyl ketone (MIBK) for 60 s. E-beam evaporation of Ti (5 nm) and Au (25 nm) layers is then performed. The sample is then placed in acetone for 6 hours for liftoff. We next remove graphene from the areas surrounding the active region of the device. This is done as follows: PMMA is spun on the sample and e-beam lithography is performed. The sample is then dry etched in an oxygen plasma. The PMMA is then removed by using acetone. We next add contact pads using e-beam lithography (with PMMA as resist) and e-beam evaporation of Ti (20 nm) and Au (100 nm) layers. This results in the graphene being connected in a field effect transistor (FET) configuration, with the Si substrate acting as the back-gate electrode. An optical microscope image of the completed sample is shown in Fig. 2a. Figure S8a,b are optical microscope images of CVD graphene transferred onto SiO_2_/Si and Al_2_O_3_/TiO_2_/Au/Si substrates, respectively. Raman spectra of these chips are provided as Fig. S8c,d, with the main peaks labelled.

### Optical characterization

Infrared reflection spectra are measured using a Fourier transform infrared spectrometer (FTIR) coupled to a microscope (Spotlight 200i, Perkin Elmer) incorporating a liquid-nitrogen-cooled mercury cadmium telluride (MCT) detector. No polarizers are used in the measurements. The variable aperture of the FTIR microscope is used to ensure that it measures the light reflected from the sample from a region that is smaller than the active region of the sample. All optical measurements are performed in ambient air.

### Spectral reconstruction

As mentioned above, our system can be modelled as $$S(\lambda )=G(\lambda )\times MC{T}_{resp}(\lambda )\times $$$${R}_{VG}(\lambda )$$.

In the spectral reconstruction problem, *S* and *R*_*VG*_ are the known values while the unknown *X* is the MCT signal $G(\lambda )\times MC{T}_{resp}$. Therefore, the problem can be classified as an inverse problem. Here we use the recursive least squares (RLS) method. This approach recursively solves an inverse problem and aims to minimize a weighted linear least squares cost function.

The algorithm starts with an initial guess of *X*. For simplicity, here we initialize *X* as a null vector. For the *i*^*th*^ recursive step, the *i*^*th*^ reflection function *R*_*VGi*_ (*λ*)_2751 × 1_ (i.e. *i*^*th*^ column of *R*_*VG*_(*λ*)_2751 × 63_ and signal data (i.e. *i*^*th*^ entry of ${S}_{1\times 63}$) are input into the algorithm. The recursive solution (*X*_*i*_) at the *i*^*th*^ step is:

$${X}_{i}={X}_{i-1}+{P}_{i}{R}_{V{G}_{i}}({S}_{i}-{R}_{V{G}_{i}}^{T}{X}_{i-1})$$

where

$${P}_{i}=[{P}_{i-1}-{P}_{i-1}{R}_{V{G}_{i}}{(\delta I+{R}_{V{G}_{i}}^{T}{P}_{i-1}{R}_{V{G}_{i}})}^{-1}{R}_{V{G}_{i}}^{T}{P}_{i-1}]{\delta }^{-1}$$

where ${R}_{V{G}_{i}}^{T}$ is the transpose of the *i*^*th*^ column of reflection function matrix ${R}_{VG}{(\lambda )}_{2751\times 63}$, *P*_*i*_ is the RLS covariance matrix and *δ* is the ‘forget factor’ $(0 < \delta \le 1)$. *P*_0_ is initialized as an identity matrix.

Within each iteration, the feedback gain term $({P}_{i}{R}_{V{G}_{i}})$ is updated to calculate an updated estimation of the *X*_*i*_. The recursive method is repeated until all data (i.e. ${R}_{VG}{(\lambda )}_{2751\times 63}$ and ${S}_{1\times 63}$) has been input to the algorithm. The final *X* is the algorithm’s estimate of the incident spectrum times the MCT detector responsivity. The detailed derivation of the RLS method could be found in ref. ^42^.

## Supplementary information

Supplementary Information.
