# Supplementary material for: Mid- to long-wave infrared computational spectroscopy with a graphene metasurface modulator
Source: Sci Rep. 2020 Mar 25;10:5377. doi: 10.1038/s41598-020-61998-w (PMC7096524; doi:10.1038/s41598-020-61998-w)
Supplement: Supplementary file 1 — Supplementary Information. [file 41598_2020_61998_MOESM1_ESM.docx]

Supporting Information

Mid- to long-wave infrared computational spectroscopy with a graphene metasurface modulator

Vivek Raj Shrestha^1^, Benjamin Craig^1^, Jiajun Meng^2^, James Bullock^2^,

Ali Javey^3,4^, and Kenneth B. Crozier^1,2^

1 School of Physics, University of Melbourne, VIC 3010, Australia

2 Department of Electrical and Electronic Engineering, University of Melbourne, VIC 3010, Australia

3 Electrical Engineering and Computer Sciences, University of California, Berkeley, Berkeley, CA 94720, USA

4 Materials Sciences Division, Lawrence Berkeley National Laboratory, Berkeley, CA 94720, USA

Corresponding Author e-mail address: kenneth.crozier@unimelb.edu.au

***
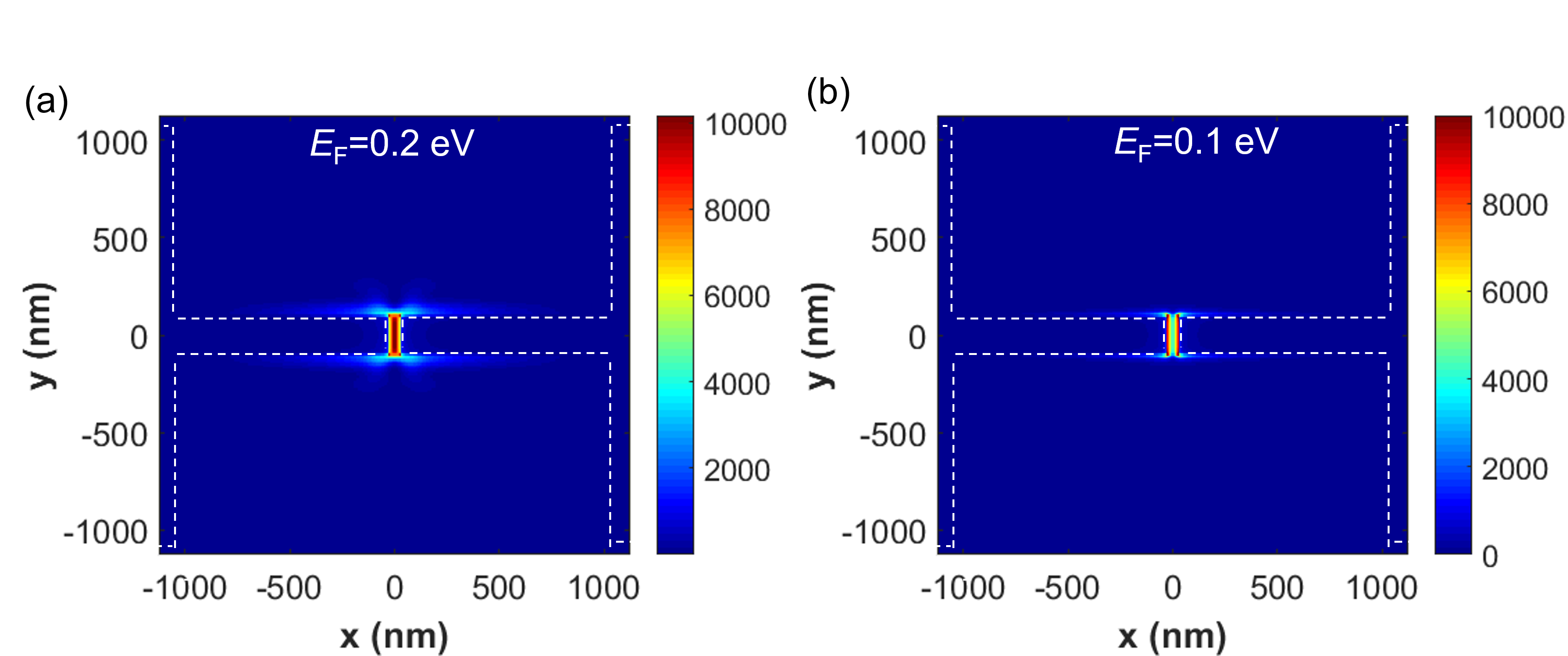
*Figure S1.** Simulated electric field intensity enhancement ${|E/E_{0}|}^{2}$ in $xy$-plane, at position of graphene, for Fermi energies of (a) 0.2 and (b) 0.1 eV. Illumination is plane wave from air-side, at normal incidence and at a wavelength of λ=9009 nm. The incident light is polarized along x-axis in the simulations.


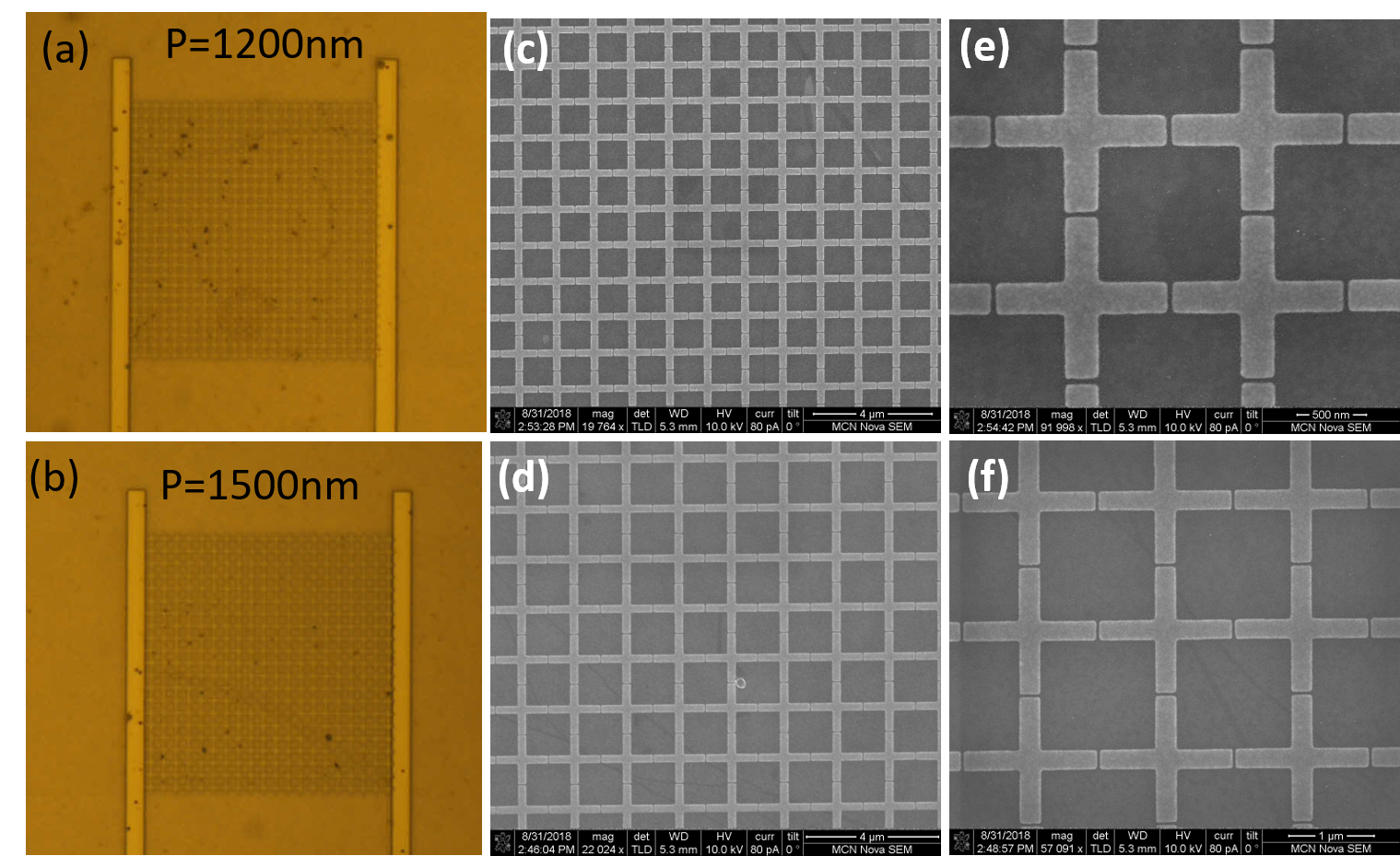


Figure S2. Optical microscope images of devices with plasmonic antennas of periods (a) $\boldsymbol{P=1200 nm}$ and (b) $\boldsymbol{P=1500 nm}$. Scanning electron microscope (SEM) images of devices with (c) $\boldsymbol{P=1200 nm}$ and (d) $\boldsymbol{P=1500 nm}$ and (e-f) their corresponding magnified SEM images.





**Figure S3.** Colormap plot of reflection spectra of devices with (a)$P= 1200 nm$ and (b) $P=1500 nm$ as a function of gate voltage from $V_{G} =-10 V$ to $10 V$, in steps of $1 V$. Measured reflection spectra of the sample with (c) $P= 1200 nm$ and (d) $P=1500 nm$ at $V_{G} =-10 V$ and $10 V$.





**Figure S4.** Experimentally measured modulation depth vs wavelength for metasurface devices (with different periods $P$).

**

**

**Figure S5.** (a) Integrated signal vs gate voltage for three metasurface devices. This figure is also included in the main text as Fig. 4b and is repeated here for convenience. (b) Spectra of infrared light source *G(λ)*, as reconstructed using single graphene metasurface modulator (red curve, with $P=2250 nm$) and as measured directly by FTIR (black curve). Spectral range for reconstruction is $\lambda=4.5\mu m$ to $10 \mu m$, with 2751 data points.. (c). Same as for panel b, but two devices ($P=2250 nm$ and $P=1500 nm$) are employed. (d) Same as for panels b and c, but three devices ($P=2250 nm$, $P=1500 nm$ and $P=1200 nm$) are employed.

The I_DS_-V_G_ graph of a representative device as the gate voltage was swept from negative (-10V) to positive (+10V) and back, indicated by the arrows denoting the sweeping direction, is displayed in Figure 1. The device showed some hysteresis and the charge neutral point (CNP) was shifted from -0.5 to 2.1V when the direction of sweep was reversed. For comparison, the shift of CNP was 1V for gate voltage sweep range in case of 300 nm SiO_2_ as gate dielectric.^S1^





**Figure S6.** I_DS_-V_G_ curve at different voltage sweep directions, where forward direction (negative to positive) is indicated by black arrow and the reverse direction (positive to negative) is indicated by red arrow.

***

***

**Figure S7.** (a) Spectra of infrared light source *G(λ)*, as reconstructed using single graphene metasurface modulator (red curve, $P=2250 nm$ device) and as measured directly by FTIR (black curve). Spectral reconstruction is performed over the wavelength range $\lambda=7.5 to 10 \mu m$with 1251 data points. Data used in spectral reconstruction is collected on same day. (b). Same as for panel a, except that the signal vs gate voltage is measured several months (150 days) later. Reconstruction is performed using reflection spectra $R\left( V_{G},\lambda\right)$ measured earlier (same data set as used in panel a).


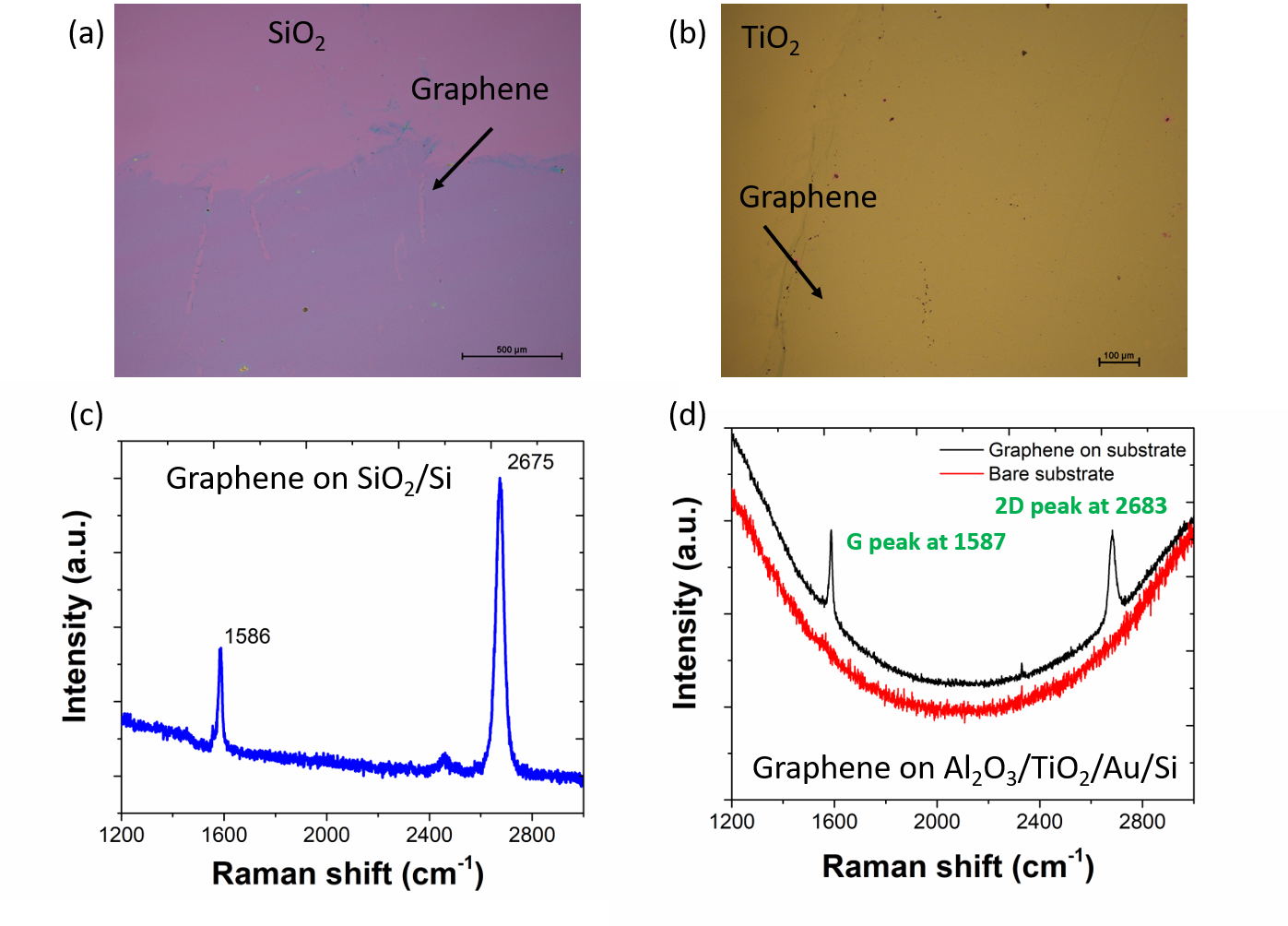
 **Figure S8.** Microscopy images of CVD graphene transferred on (a) SiO_2_|Si substrate (b) and Al2O3/TiO2/Au/Si substrate and corresponding Raman spectrum are shown in (c) and (d) respectively with the main peaks labeled.

S1. Wang, H., Wu, Y., Cong, C., Shang, J. & Yu, T. Hysteresis of Electronic Transport in Graphene Transistors. ACS Nano 4, 7221-7228, doi:10.1021/nn101950n (2010).
